# Generalized disequilibrium test for association in qualitative traits incorporating imprinting effects based on extended pedigrees

**DOI:** 10.1186/s12863-017-0560-0

**Published:** 2017-10-16

**Authors:** Jian-Long Li, Peng Wang, Wing Kam Fung, Ji-Yuan Zhou

**Affiliations:** 1 State Key Laboratory of Organ Failure Research, Ministry of Education, and Guangdong Provincial Key Laboratory of Tropical Disease Research, Department of Biostatistics, School of Public Health, Southern Medical University, Guangzhou, China; 2 State Key Laboratory of Ophthalmology, Zhongshan Ophthalmic Center, Sun Yat-sen University, Guangzhou, China; 3 Department of Statistics and Actuarial Science, The University of Hong Kong, Hong Kong, China

**Keywords:** Generalized disequilibrium test, Genomic imprinting, Monte Carlo sampling, Qualitative trait

## Abstract

**Background:**

For dichotomous traits, the generalized disequilibrium test with the moment estimate of the variance (GDT-ME) is a powerful family-based association method. Genomic imprinting is an important epigenetic phenomenon and currently, there has been increasing interest of incorporating imprinting to improve the test power of association analysis. However, GDT-ME does not take imprinting effects into account, and it has not been investigated whether it can be used for association analysis when the effects indeed exist.

**Results:**

In this article, based on a novel decomposition of the genotype score according to the paternal or maternal source of the allele, we propose the generalized disequilibrium test with imprinting (GDTI) for complete pedigrees without any missing genotypes. Then, we extend GDTI and GDT-ME to accommodate incomplete pedigrees with some pedigrees having missing genotypes, by using a Monte Carlo (MC) sampling and estimation scheme to infer missing genotypes given available genotypes in each pedigree, denoted by MCGDTI and MCGDT-ME, respectively. The proposed GDTI and MCGDTI methods evaluate the differences of the paternal as well as maternal allele scores for all discordant relative pairs in a pedigree, including beyond first-degree relative pairs. Advantages of the proposed GDTI and MCGDTI test statistics over existing methods are demonstrated by simulation studies under various simulation settings and by application to the rheumatoid arthritis dataset. Simulation results show that the proposed tests control the size well under the null hypothesis of no association, and outperform the existing methods under various imprinting effect models. The existing GDT-ME and the proposed MCGDT-ME can be used to test for association even when imprinting effects exist. For the application to the rheumatoid arthritis data, compared to the existing methods, MCGDTI identifies more loci statistically significantly associated with the disease.

**Conclusions:**

Under complete and incomplete imprinting effect models, our proposed GDTI and MCGDTI methods, by considering the information on imprinting effects and all discordant relative pairs within each pedigree, outperform all the existing test statistics and MCGDTI can recapture much of the missing information. Therefore, MCGDTI is recommended in practice.

**Electronic supplementary material:**

The online version of this article (10.1186/s12863-017-0560-0) contains supplementary material, which is available to authorized users.

## Background

Genomic imprinting is an important epigenetic phenomenon in studying complex traits, where the expression levels of certain genes rely on their parental origin [1–3]. Morison et al. [4, 5] constructed an imprinted gene and parent-of-origin effect database to collect genes that show imprinting effects, which has been updated by Glaser et al. [6] to include parental origin of de novo mutations. Furthermore, some researches have demonstrated that genomic imprinting plays an important role in several human genetic diseases such as Beckwith-Wiedemann syndrome, Silver-Russell syndrome, pseudohypoparathyroidism and transient neonatal diabetes mellitus [7–10].

For a diallelic marker locus, there have been many family-based methods to test for the association between genotype scores and dichotomous traits [11–15]. Among them, the generalized disequilibrium test with the moment estimate of the variance (GDT-ME) [15] is a powerful method, which is the generalization of the traditional transmission disequilibrium test [11] by using the genotype differences between all discordant relative pairs (including those beyond first-degree relatives) within a family. Currently, there has been increasing interest of incorporating imprinting to improve the test power of association analysis. However, GDT-ME does not take imprinting effects into account, and it has not been investigated whether it can be used for association analysis when the effects indeed exist. On the other hand, Xia et al. [16] developed the transmission disequilibrium test with imprinting for qualitative traits based on two-generation nuclear families, while it is not suitable for extended pedigrees. As such, the pedigree disequilibrium test with imprinting (PDTI) and its extension Monte Carlo (MC) PDTI (MCPDTI) to accommodate pedigrees with missing genotypes were proposed to test for association, which consider the influence of imprinting on association study [17]. However, they only utilize the genotype differences between all first-degree relative pairs in a family, which may reduce their test powers if ignoring the information on the genotype differences between beyond first-degree relatives.

To incorporate imprinting effects into association analysis, in this article, we develop a novel decomposition of the genotype score of each individual according to the paternal or maternal source of the allele. Based on these paternal and maternal allele scores, we propose the generalized disequilibrium test with imprinting (GDTI) for association for complete pedigrees without any missing genotypes. Then, borrowing the idea of Zhou et al. [18] and Ding et al. [19], we further extend GDTI and GDT-ME to accommodate incomplete pedigrees where the genotypes of some individuals in pedigrees are missing, based on a MC sampling and estimation scheme to infer the missing genotypes given the observed genotypes in each pedigree, which are denoted by MCGDTI and MCGDT-ME, respectively. Advantages of the proposed GDTI and MCGDTI test statistics over existing methods are demonstrated by simulation studies under various simulation settings and by application to the rheumatoid arthritis (RA) dataset [20]. Simulation results show that the proposed GDTI, MCGDTI and MCGDT-ME control the type I error rates well under the null hypothesis of no association and no imprinting. The existing GDT-ME and the proposed MCGDT-ME can be used to test for association even when imprinting effects exist. MCGDTI can recapture much of the missing information. Further, the proposed tests outperform the existing methods under complete, incomplete and no imprinting effect models. For the real data application, compared to the existing methods, MCGDTI identifies more loci statistically significantly associated with RA after Bonferroni correction.

## Methods

### Notations

Suppose a diallelic marker locus with alleles *M*_1_ and *M*_2_, and three possible genotypes are respectively *M*_2_ *M*_2_, *M*_1_ *M*_2_ and *M*_1_ *M*_1_. We consider a disease susceptibility locus with the disease allele *D* and the normal one *d*, and the corresponding ordered genotypes are *D*/*D*, *D*/*d*, *d*/*D* and *d*/*d* with penetrances *f*_2_, *f*_10_, *f*_01_ and *f*_0_, respectively. *f*_10_ = *f*_01_ indicates no imprinting effects at the disease susceptibility locus. Further, the coefficient of linkage disequilibrium (LD) between alleles *M*_1_ and *D* is taken as $\mathrm{LD}=P\left(D{M}_1\right)-{P}_D{P}_{M_1}$, where *P*(*DM*_1_) is the frequency of haplotype *DM*_1_, and *P*_*D*_ and ${P}_{M_1}$ are the allele frequency of *D* and *M*_1_, respectively. Suppose that we collect *n* independent pedigrees. Within the *i* ^th^ pedigree which contains *N*_*i*_ family members (*i*=1, 2, …, *n*), without loss of generality, we assume that the first *A*_*i*_individuals are affected and the other *U*_*i*_ = *N*_*i*_ − *A*_*i*_ members are unaffected. Let *Y*_*ij*_ be the disease status of the *j* ^th^ individual in the *i* ^th^ pedigree (*i*=1, 2, …, *n*; *j*=1, 2, …, *N*_*i*_), i.e. *Y*_*ij*_= 1 (0) denotes that the individual is affected (unaffected).

### Existing generalized disequilibrium test with moment estimate of variance

We begin by describing the existing GDT-ME test [15]. For convenience, we define the genotype score *X*_*ij*_ by the number of allele *M*_1_ in the genotype of the *j* ^th^ individual in the *i* ^th^ pedigree, i.e. *X*_*ij*_=0, 1 and 2 for the genotypes *M*_2_ *M*_2_, *M*_1_ *M*_2_ and *M*_1_ *M*_1_, respectively. As such, the logistic regression model is

1 $$ \log \frac{P\left({Y}_{ij}=1\right)}{1-P\left({Y}_{ij}=1\right)}={\beta}_0+{\beta}_1{X}_{ij}, $$

where *β*_0_ is the intercept, and *β*_1_ is the regression coefficient; *Y*_*ij*_ is the disease status of the *j* ^th^ individual in the *i* ^th^ pedigree. Then, the GDT-ME test statistic can be expressed as follows, which is used to model the association between the disease status and *X*_*ij*_:

2 $$ \mathrm{GDT}-\mathrm{ME}=\frac{\sum_{i=1}^n{S}_i}{\sqrt{\sum_{i=1}^n{S}_i^2}}=\frac{\sum_{i=1}^n\sum_{j=1}^{A_i}\sum_{k={A}_i+1}^{N_i}\left({X}_{ij}-{X}_{ik}\right)\frac{1}{N_i}}{\sqrt{\sum_{i=1}^n{\left(\sum_{j=1}^{A_i}\sum_{k={A}_i+1}^{N_i}\left({X}_{ij}-{X}_{ik}\right)\frac{1}{N_i}\right)}^2}}, $$

where ${S}_i=\sum_{j=1}^{A_i}\sum_{k={A}_i+1}^{N_i}\left({X}_{ij}-{X}_{ik}\right)\frac{1}{N_i}$ is the score of the *i* ^th^ pedigree and ${\sum}_{i=1}^n{S}_i^2$ is an unbiased moment estimate of the variance of $\sum_{i=1}^n{S}_i$. The variance of $\sum_{i=1}^n{S}_i$ can also be estimated based on the information on kinship coefficients when identity by descent (IBD) is unknown [15]. For convenience, we denote the corresponding test statistic by GDT in this article.

### GDTI for complete pedigree data

Although GDT-ME is a powerful association test and is robust to population stratification (PS) [15], it does not take the information on imprinting effects into consideration. In this article, we are going to investigate whether GDT-ME can be used to test for association when there are imprinting effects. Moreover, we propose the following generalized disequilibrium test incorporating imprinting effects (GDTI). Note that in GDT-ME, the genotype score *X*_*ij*_ is coded as the counts of allele *M*_1_ for the *j* ^th^ individual in the *i* ^th^ pedigree, i.e.

$$ {X}_{ij}=\left\{\begin{array}{c}0,\kern2em {M}_2{M}_2\ \\ {}1,\kern2em {M}_1{M}_2\ \\ {}2,\kern2em {M}_1{M}_1\ \end{array}\right.. $$

To incorporate the information on imprinting effects into analysis, we divide the *X*_*ij*_ into two parts, ${X}_{ij}^{(p)}$ and ${X}_{ij}^{(m)}$, according to the paternal or maternal source of the allele, where ${X}_{ij}={X}_{ij}^{(p)}+{X}_{ij}^{(m)}$, and ${X}_{ij}^{(p)}$ and ${X}_{ij}^{(m)}$ are respectively coded as follows:

$$ {X}_{ij}^{(p)}=\left\{\begin{array}{c}0,\kern0.5em \mathrm{if}\  \mathrm{the}\ {\mathrm{individual}}^{\hbox{'}}\mathrm{s}\ \mathrm{genotype}\  \mathrm{is}\ {M}_2{M}_2,\mathrm{which}\  \mathrm{indicates}\  \mathrm{thatone}\\ {}\ \mathrm{of}\ \mathrm{two}\ {M_2}^{\hbox{'}}\mathrm{s}\ \mathrm{came}\  \mathrm{from}\  \mathrm{father},\mathrm{or}\ {M}_1{M}_2\ \mathrm{with}\ {M}_1\mathrm{coming}\  \mathrm{from}\\ {}\ \mathrm{mother}\kern22.5em \\ {}0.5,\kern0.4em \mathrm{if}\  \mathrm{the}\ {\mathrm{individual}}^{\hbox{'}}\mathrm{s}\ \mathrm{genotype}\  \mathrm{is}\ {M}_1{M}_2,\mathrm{but}\ \mathrm{it}\ \mathrm{is}\ \mathrm{not}\ \mathrm{s}\mathrm{urewhich}\\ {}\ \mathrm{allele}\  \mathrm{came}\  \mathrm{from}\  \mathrm{father}\ \Big(\mathrm{i}.\mathrm{e}.\mathrm{the}\ {M}_1{M}_2\ \mathrm{founders},\mathrm{or}\  \mathrm{the}\ {M}_1{M}_2\\ {}\ \mathrm{nonfounders}\  \mathrm{with}\  \mathrm{both}\  \mathrm{parents}\  \mathrm{beingheterozygous}\Big)\\ {}1,\kern0.4em \mathrm{if}\  \mathrm{the}\ {\mathrm{individual}}^{\hbox{'}}\mathrm{s}\ \mathrm{genotype}\  \mathrm{is}\ {M}_1{M}_1,\mathrm{or}\ {M}_1{M}_2\ \mathrm{with}\ {M}_1\mathrm{coming}\\ {}\ \mathrm{from}\  \mathrm{father}\end{array}\right., $$

and

$$ {X}_{ij}^{(m)}=\left\{\begin{array}{c}0,\mathrm{if}\  \mathrm{the}\ {\mathrm{individual}}^{\hbox{'}}\mathrm{s}\ \mathrm{genotype}\  \mathrm{is}\ {M}_2{M}_2,\mathrm{whichindicates}\  \mathrm{that}\ \mathrm{one}\\ {}\ \mathrm{of}\ \mathrm{two}\ {M_2}^{\hbox{'}}\mathrm{s}\ \mathrm{came}\  \mathrm{from}\mathrm{mother},\mathrm{or}\ {M}_1{M}_2\ \mathrm{with}\ {M}_1\ \mathrm{coming}\  \mathrm{from}\\ {}\ \mathrm{father}\\ {}0.5,\mathrm{if}\  \mathrm{the}\ {\mathrm{individual}}^{\hbox{'}}\mathrm{s}\ \mathrm{genotype}\  \mathrm{is}\ {M}_1{M}_2,\mathrm{but}\ \mathrm{it}\ \mathrm{is}\  \mathrm{notsure}\  \mathrm{which}\\ {}\ \mathrm{allele}\  \mathrm{came}\  \mathrm{from}\  \mathrm{mother}\Big(\mathrm{i}.\mathrm{e}.\mathrm{the}\ {M}_1{M}_2\ \mathrm{founders},\mathrm{or}\  \mathrm{the}\ {M}_1{M}_2\\ {}\ \mathrm{nonfounderswith}\  \mathrm{both}\  \mathrm{parents}\  \mathrm{being}\  \mathrm{heterozygous}\Big)\\ {}1,\mathrm{if}\  \mathrm{the}\ {\mathrm{individual}}^{\hbox{'}}\mathrm{s}\ \mathrm{genotype}\  \mathrm{is}\ {M}_1{M}_1,\mathrm{or}\ {M}_1{M}_2\mathrm{with}\ {M}_1\ \mathrm{coming}\\ {}\ \mathrm{from}\  \mathrm{mother}\end{array}\right.. $$

We call ${X}_{ij}^{(p)}$ and ${X}_{ij}^{(m)}$ the paternal allele score and the maternal allele score, respectively. So, we use the following logistic regression to model the association between the disease status *Y*_*ij*_ and the allele scores ${X}_{ij}^{(p)}$ and ${X}_{ij}^{(m)}$:

$$ \mathit{\log}\frac{P\left({Y}_{ij}=1\right)}{1-P\left({Y}_{ij}=1\right)}={\beta}_0+{\beta}_p{X}_{ij}^{(p)}+{\beta}_m{X}_{ij}^{(m)}, $$

where *β*_0_ is the intercept, and *β*_*p*_ and *β*_*m*_ are the regression coefficients; *β*_*p*_ is used to describe the effect of allele *M*_1_ coming from his (her) father, and *β*_*m*_ measures the effect of allele *M*_1_ whose parental origin is his (her) mother. The null hypothesis *H*_0_ : *β*_*p*_ = *β*_*m*_ = 0 denotes no association and no imprinting; *β*_*p*_ = *β*_*m*_ ≠ 0 indicates that the association exists while there are no imprinting effects, and the logistic regression model can be reduced to the model of GDT-ME (Equation (1)); *β*_*p*_ ≠ *β*_*m*_ represents that both association and imprinting effects exist. As such,

$$ P\left({Y}_{ij}=1\right)=\frac{\exp \left({\beta}_0+{\beta}_p{X}_{ij}^{(p)}+{\beta}_m{X}_{ij}^{(m)}\right)}{1+\exp \left({\beta}_0+{\beta}_p{X}_{ij}^{(p)}+{\beta}_m{X}_{ij}^{(m)}\right)}. $$

Note that the disease statuses of all the family members in each pedigree are uncorrelated, conditional on their own genotypes at the marker locus. Then, the likelihood that the first *A*_*i*_ individuals are affected, conditional on the fact that there are *A*_*i*_ affected individuals in total in the *i* ^th^ pedigree, is (the detailed derivation refers to Additional file 1: Appendix):

$$ P\left(\sum_{j=1}^{A_i}{Y}_{ij}={A}_i|\sum_{j=1}^{N_i}{Y}_{ij}={A}_i\right)=\frac{P\left(\sum_{j=1}^{A_i}{Y}_{ij}={A}_i\right)}{\sum_{s_l}P\left({\sum}_{j\epsilon {s}_l}{Y}_{ij}={A}_i\right)}=\frac{\mathit{\exp}\left\{\frac{1}{U_i}\sum_{j=1}^{A_i}\sum_{k={A}_i+1}^{N_i}\left[\left({X}_{ij}^{(p)}-{X}_{ik}^{(p)}\right){\beta}_p+\left({X}_{ij}^{(m)}-{X}_{ik}^{(m)}\right){\beta}_m\right]\right\}}{\sum_{s_l}\mathit{\exp}\left\{\frac{1}{U_i}\sum_{j\epsilon {s}_l}\sum_{k={A}_i+1}^{N_i}\left[\left({X}_{ij}^{(p)}-{X}_{ik}^{(p)}\right){\beta}_p+\left({X}_{ij}^{(m)}-{X}_{ik}^{(m)}\right){\beta}_m\right]\right\}}, $$

where *s*_*l*_’s are all the possible combination that *A*_*i*_ out of *N*_*i*_ individuals are affected by shuffling the affection statuses of all the *N*_*i*_ individuals in the *i* ^th^ pedigree; *s*_*l*_ is the *l* ^th^ possible combination; *U*_*i*_ = *N*_*i*_ − *A*_*i*_ is the number of unaffected individuals in the *i* ^th^ pedigree. As such, the log-likelihood function for the *i* ^th^ pedigree is

$$ {l}_i=\frac{1}{U_i}\sum_{j=1}^{A_i}\sum_{k={A}_i+1}^{N_i}\left[\left({X}_{ij}^{(p)}-{X}_{ik}^{(p)}\right){\beta}_p+\left({X}_{ij}^{(m)}-{X}_{ik}^{(m)}\right){\beta}_m\right]-\log \sum_{s_l}\exp \left(\frac{1}{U_i}\sum_{j\epsilon {s}_l}\sum_{k={A}_i+1}^{N_i}\left[\left({X}_{ij}^{(p)}-{X}_{ik}^{(p)}\right){\beta}_p+\left({X}_{ij}^{(m)}-{X}_{ik}^{(m)}\right){\beta}_m\right]\right). $$

Under the null hypothesis of no association (*H*_0_ : *β*_*p*_ = *β*_*m*_ = 0), the score test statistic for testing for association incorporating imprinting effects is formulated as follows (the details see Additional file 1: Appendix),

3 $$ \mathrm{GDTI}=\left(\sum_{i=1}^n{D}_{i1}\kern0.5em \sum_{i=1}^n{D}_{i2}\right)\bullet {\left(\begin{array}{cc}\sum \limits_{i=1}^n{I}_{i11}& \sum \limits_{i=1}^n{I}_{i12}\\ {}\sum \limits_{i=1}^n{I}_{i21}& \sum \limits_{i=1}^n{I}_{i22}\end{array}\right)}^{-1}\bullet \left(\begin{array}{cc}{\sum}_{i=1}^n& {\mathrm{D}}_{i1}\\ {}{\sum}_{i=1}^n& {\mathrm{D}}_{i2}\end{array}\right), $$

where $\sum_{i=1}^n{D}_{i1}$ and $\sum_{i=1}^n{D}_{i2}$ are the scores of *β*_*p*_ and *β*_*m*_, respectively;

$$ \left(\begin{array}{cc}\sum \limits_{i=1}^n{I}_{i11}& \sum \limits_{i=1}^n{I}_{i12}\\ {}\sum \limits_{i=1}^n{I}_{i21}& \sum \limits_{i=1}^n{I}_{i22}\end{array}\right) $$ is the observed Fisher’s information matrix of *β*_*p*_ and *β*_*m*_;

$$ {D}_{i1}=\frac{1}{N_i}\sum_{j=1}^{A_i}\sum_{k={A}_i+1}^{N_i}\left({X}_{ij}^{(p)}-{X}_{ik}^{(p)}\right),\kern1em {D}_{i2}=\frac{1}{N_i}\sum_{j=1}^{A_i}\sum_{k={A}_i+1}^{N_i}\left({X}_{ij}^{(m)}-{X}_{ik}^{(m)}\right), $$

$$ {I}_{i11}=-{\left.\frac{\partial^2{l}_i}{\partial {\beta}_p^2}\right|}_{\beta_p=0,{\beta}_m=0\kern0.5em }=\frac{{\left(\frac{1}{U_i}\right)}^2\sum_{s_l}{\left[\sum_{j\epsilon {s}_l}\sum_{k={A}_i+1}^{N_i}\left({X}_{ij}^{(p)}-{X}_{ik}^{(p)}\right)\right]}^2}{\left(\genfrac{}{}{0pt}{}{N_i}{A_i}\right)}-{\left(\frac{A_i}{U_i{N}_i}\right)}^2{\left[\sum_{j=1}^{A_i}\sum_{k={A}_i+1}^{N_i}\left({X}_{ij}^{(p)}-{X}_{ik}^{(p)}\right)\right]}^2, $$

$$ {I}_{i22}=-{\left.\frac{\partial^2{l}_i}{\partial {\beta}_m^2}\right|}_{\beta_p=0,{\beta}_m=0\kern0.5em }=\frac{{\left(\frac{1}{U_i}\right)}^2\sum_{s_l}{\left[\sum_{j\epsilon {s}_l}\sum_{k={A}_i+1}^{N_i}\left({X}_{ij}^{(m)}-{X}_{ik}^{(m)}\right)\right]}^2}{\left(\genfrac{}{}{0pt}{}{N_i}{A_i}\right)}-{\left(\frac{A_i}{U_i{N}_i}\right)}^2{\left[\sum_{j=1}^{A_i}\sum_{k={A}_i+1}^{N_i}\left({X}_{ij}^{(m)}-{X}_{ik}^{(m)}\right)\right]}^2, $$

and

$$ {I}_{i12}={I}_{i21}=-{\left.\frac{\partial^2{l}_i}{\partial {\beta}_p\partial {\beta}_m}\right|}_{\beta_p=0,{\beta}_m=0\kern0.5em } $$

$$ =\frac{{\left(\frac{1}{U_i}\right)}^2\sum_{s_l}\left\{\left[\sum_{j\epsilon {s}_l}\sum_{k={A}_i+1}^{N_i}\left({X}_{ij}^{(p)}-{X}_{ik}^{(p)}\right)\right]\left[\sum_{j\epsilon {s}_l}\sum_{k={A}_i+1}^{N_i}\left({X}_{ij}^{(m)}-{X}_{ik}^{(m)}\right)\right]\right\}}{\left(\genfrac{}{}{0pt}{}{N_i}{A_i}\right)}-{\left(\frac{A_i}{U_i{N}_i}\right)}^2\left[\sum_{j=1}^{A_i}\sum_{k={A}_i+1}^{N_i}\left({X}_{ij}^{(p)}-{X}_{ik}^{(p)}\right)\right]\left[\sum_{j=1}^{A_i}\sum_{k={A}_i+1}^{N_i}\left({X}_{ij}^{(m)}-{X}_{ik}^{(m)}\right)\right]. $$

GDTI asymptotically follows a chi-square distribution with the degrees of freedom being 2, under the null hypothesis of no association and no imprinting. It is noted from the above that the scores *D*_*i*1_ and *D*_*i*2_evaluate the differences in paternal allele scores and maternal allele scores, respectively, for all discordant relative pairs in a pedigree, thus utilizing information beyond first-degree relative pairs. This is in contrast to other association testing methods under imprinting (e.g. PDTI), where extended pedigrees are considered as multiple nuclear families, and so information is not fully utilized.

### MCGDTI and MCGDT-ME for incomplete pedigree data

When the genotypes of some individuals in a pedigree are missing, GDTI cannot be used directly. Therefore, in presence of missingness, we extend GDTI and propose MCGDTI based on a MC sampling and estimation process, which may recapture most information on missing genotypes based on the observed genotypes. Specifically, we replace *D*_*i*1_, *D*_*i*2_, *I*_*i*11_, *I*_*i*12_, *I*_*i*21_ and *I*_*i*22_ in GDTI by their conditional expectations, *D*_*i*1*MC*_, *D*_*i*2*MC*_, *I*_*i*11*MC*_, *I*_*i*12*MC*_, *I*_*i*21*MC*_ and *I*_*i*22*MC*_, given the observed genotypes, *G*_*o*_, where *T*_*MC*_ = *E*(*T*(*G*_*m*_, *G*_*o*_, *A*)| *G*_*o*_) for some statistic *T*, *G*_*m*_ is the set of missing genotypes; *A* is the collection of the observed phenotypes (disease affection statuses); *T*(*G*_*m*_, *G*_*o*_, *A*) is the expanded notation of *T* to explicitly show its dependences on the missing genotypes *G*_*m*_, the observed genotypes *G*_*o*_ and the observed phenotype collection *A*. Following Zhou et al. [18] and Ding et al. [19], we estimate *D*_*i*1*MC*_, *D*_*i*2*MC*_, *I*_*i*11*MC*_, *I*_*i*12*MC*_, *I*_*i*21*MC*_ and *I*_*i*22*MC*_ based on a MC simulation scheme. Specifically, if we set the MC size to be *K*, then we draw independent sample *G*_*mk*_, *k* = 1, 2, …, *K*, from *P*(*G*_*m*_| *G*_*o*_), which can be accomplished efficiently based on the peeling algorithm using the SLINK software [21]. The statistic *D*_*i*1*MC*_ can be estimated by${\widehat{D}}_{i1 MC}=\frac{1}{K}\sum_{k=1}^K{D}_{i1}\left({G}_{mk},{G}_o,A\right)$. *D*_*i*2*MC*_, *I*_*i*11*MC*_, *I*_*i*12*MC*_, *I*_*i*21*MC*_ and *I*_*i*22*MC*_ can be similarly estimated by ${\widehat{D}}_{i2 MC}$, ${\widehat{I}}_{i11 MC}$, ${\widehat{I}}_{i12 MC}$,${\widehat{I}}_{i21 MC}$ and ${\widehat{I}}_{i22 MC}$, respectively. Then, the MCGDTI statistic is calculated after replacing *D*_*i*1_, *D*_*i*2_, *I*_*i*11_, *I*_*i*12_, *I*_*i*21_ and *I*_*i*22_ in Equation (3) by the corresponding ${\widehat{D}}_{i1 MC}$, ${\widehat{D}}_{i2 MC}$, ${\widehat{I}}_{i11 MC}$, ${\widehat{I}}_{i12 MC}$, ${\widehat{I}}_{i21 MC}$ and ${\widehat{I}}_{i22 MC}$ values, respectively. MCGDTI has an asymptotic chi-square distribution with the degrees of freedom being 2 under the null hypothesis.

Earlier studies showed that the transmission disequilibrium test can be employed for association analysis even when there are imprinting effects [16], and we find out that GDT-ME can also be used for such a purpose (see simulation studies later). In this connection, for incomplete pedigree data, we extend GDT-ME without considering imprinting effects and propose MCGDT-ME to test for association based on the MC sampling and estimation scheme. Being similar to MCGDTI, the MCGDT-ME statistic can be calculated, as before, but substituting each *S*_*i*_ in Equation (2) by ${S}_{iMC}=\frac{1}{K}{\sum}_{k=1}^K{S}_i\left({G}_{mk},{G}_o,A\right)$, i.e. MCGDT-ME$=\sum_{i=1}^n{S}_{iMC}/\sqrt{\sum_{i=1}^n{S}_{iMC}^2}$. MCGDT-ME follows a standard normal distribution approximately under the null hypothesis of no association.

### Simulation settings

In this section, to explore the performance of the proposed GDTI, MCGDTI and MCGDT-ME statistics and compare the powers of GDTI, MCGDTI and MCGDT-ME with the existing MCPDTI, GDT-ME and GDT, we conduct the following simulation studies. We consider a homogeneous population. The marker locus and the disease susceptibility locus are in complete linkage. Three groups of haplotype frequencies for haplotypes *DM*_1_, *dM*_1_, *DM*_2_ and *dM*_2_ are considered to simulate the powers: LD1: {0.13, 0.02, 0.12, 0.73}, LD2: {0.23, 0.12, 0.02, 0.63} and LD3: {0.22, 0.03, 0.03, 0.72}, where the frequency ${P}_{M_1}$ of marker allele *M*_1_ for each group is 0.15, 0.35 and 0.25 with the frequency *P*_*D*_ of the disease allele *D* being fixed at 0.25, and the corresponding LD values are 0.092,5, 0.142,5 and 0.157,5, respectively. To investigate the empirical type I error rates under the null hypothesis of no association, the frequencies of four haplotypes are taken as the product of two allele frequencies on each haplotype, respectively. For example, when ${P}_{M_1}=0.15$, the frequency of haplotype *DM*_1_ is *P*(*DM*_1_)= 0.15×0.25 = 0.037,5.

Three sets of two homozygote penetrances *f*_2_ and *f*_0_ for genotypes *D*/*D* and *d*/*d*, {0.390, 0.260}, {0.440, 0.240} and {0.480, 0.220}, are investigated with the corresponding relative risk (RR=*f*_2_/*f*_0_) being 1.500, 1.833 and 2.182, respectively, which are similar to those in Ding et al. [19]. For each set of homozygote penetrances, three imprinting effect models by setting the various values of *f*_10_ and *f*_01_ are considered: no, incomplete and complete imprinting effect models. For no imprinting effect model, we set *f*_1_ = *f*_10_ = *f*_01_ = (*f*_2_ + *f*_0_)/2. Note that no association implies no imprinting effects. So, we simulate the type I error rates of the proposed test statistics only under no association and no imprinting. Tables 1 and 2 give the simulation settings for studying the empirical size and the test power, respectively.

**Table 1:** Simulation settings for estimating size

Setting	P_M1_	f_2_	f_1_	f_0_	RR
1	0.15	0.390	0.325	0.260	1.500
2	0.15	0.440	0.340	0.240	1.833
3	0.15	0.480	0.350	0.220	2.182
4	0.35	0.390	0.325	0.260	1.500
5	0.35	0.440	0.340	0.240	1.833
6	0.35	0.480	0.350	0.220	2.182
7	0.25	0.390	0.325	0.260	1.500
8	0.25	0.440	0.340	0.240	1.833
9	0.25	0.480	0.350	0.220	2.182

**Table 2:** Simulation settings for estimating power

A Haplotype frequencies					
LD setting	DM_1_	dM_1_	DM_2_	dM_2_	LD
LD1	0.130	0.020	0.120	0.730	0.0925
LD2	0.230	0.120	0.020	0.630	0.1425
LD3	0.220	0.030	0.030	0.720	0.1575
B Penetrances and imprinting effect models					
Imprinting effect model	f_2_	f_10_	f_01_	f_0_	RR
No	0.390	0.325	0.325	0.260	1.500
	0.440	0.340	0.340	0.240	1.833
	0.480	0.350	0.350	0.220	2.182
Incomplete	0.390	0.370	0.280	0.260	1.500
	0.440	0.420	0.260	0.240	1.833
	0.480	0.460	0.240	0.220	2.182
Complete	0.390	0.390	0.260	0.260	1.500
	0.440	0.440	0.240	0.240	1.833
	0.480	0.480	0.220	0.220	2.182

**Fig. 1:**
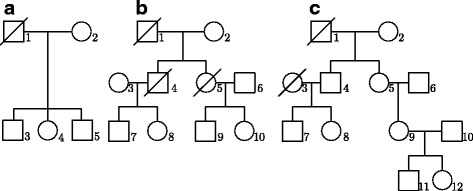
Pedigree structures for the simulation studies. **a** Two-generation family. **b** Three-generation pedigree. **c** Four-generation pedigree. Genotypes of individual 1 in two-generation family, individuals 1, 4 and 5 in three-generation pedigree and individuals 1 and 3 in four-generation pedigree are assumed to be missing for the analysis based on incomplete data

In addition, three types of pedigree structure are considered in our simulation study. The pedigree structures are shown in Fig. 1: (a) two-generation family with 5 individuals, (b) three-generation pedigree with 10 individuals, and (c) four-generation pedigree with 12 individuals. In each replicate, we simulate 30 (50) pedigrees under each pedigree structure and the resulting total sample size is 90 (150). Here the ascertainment scheme for a pedigree to be included is that there is at least one affected nonfounder in the pedigree. For MCGDTI, MCGDT-ME and MCPDTI, 50 MC samples of missing genotypes are generated for each replicate with use of the SLINK software [21]. In the MC sampling process, both the true marker allele frequencies and those estimated from the genotyped founders in each replicate are used.

For assessing the performance of the proposed tests (GDTI, MCGDTI and MCGDT-ME) and for comparing with the existing GDT-ME and GDT without considering imprinting effects [15], and MCPDTI with incorporating imprinting [17], we consider the following 9 tests. GDTI is based on complete data assuming no missing genotypes. The other 8 tests are for incomplete data, after the removal of the genotypes of individual 1 in two-generation families, individuals 1, 4 and 5 in three-generation pedigrees and individuals 1 and 3 in four-generation pedigrees. MCGDTI_T_, MCGDT-ME_T_ and MCPDTI_T_ are on the basis of the true marker allele frequencies, while MCGDTI_E_, MCGDT-ME_E_ and MCPDTI_E_ are based on the estimated marker allele frequencies. GDT-ME and GDT are also considered for incomplete data. Under each simulation setting, 10,000 replicates are simulated and the significance level is set at 1%. All the simulations are implemented by using the R software (version 3.4.1) [22].

## Results

### Size and power

Under 9 simulation settings given in Table 1, the empirical type I error rates of GDTI, MCGDTI_T_, MCGDTI_E_, MCGDT-ME_T_, MCGDT-ME_E_, GDT-ME, GDT, MCPDTI_T_ and MCPDTI_E_ are demonstrated in Table 3, based on 90 and 150 pedigrees at the 1% significance level, respectively. It is shown in Table 3 that the size of all the methods is generally close to the nominal level 1% under the null hypothesis of no association and no imprinting, irrespective of different sample sizes. Thus, our proposed GDTI, MCGDTI_T_, MCGDTI_E_, MCGDT-ME_T_ and MCGDT-ME_E_ test statistics are valid for testing association.

**Table 3:** Empirical size (in percentage (%)) of GDTI, MCGDTI, MCGDT-ME, GDT-ME, GDT and MCPDTI^a^

Setting	Complete data		Incomplete data							
	GDTI		MCGDTI_T_	MCGDTI_E_	MCGDT-ME_T_	MCGDT-ME_E_	GDT-ME	GDT	MCPDTI_T_	MCPDTI_E_
Based on 90 pedigrees										
1	0.98		1.04	1.10	0.95	0.99	0.82	0.85	0.80	0.84
2	1.14		1.12	1.14	1.12	1.06	0.82	0.87	0.66	0.74
3	0.99		1.05	0.99	0.91	0.94	0.80	0.87	0.81	0.86
4	0.85		0.97	1.05	0.98	0.91	0.74	0.90	0.91	0.91
5	0.96		1.11	1.11	0.96	0.88	0.87	1.03	0.73	0.81
6	1.11		1.13	1.14	0.84	0.81	0.98	1.05	0.90	0.89
7	1.13		0.85	0.96	0.96	0.88	0.70	0.82	0.94	1.05
8	1.09		1.11	1.13	0.86	0.86	0.72	0.76	0.83	0.86
9	1.11		1.14	1.13	1.05	1.12	0.82	0.89	1.04	1.00
Based on 150 pedigrees										
1	1.00		0.93	0.94	0.94	0.93	0.93	0.95	0.90	0.98
2	1.10		1.05	1.04	0.98	0.98	0.98	1.09	0.88	0.93
3	1.05		1.09	1.10	0.97	0.99	1.04	1.04	0.89	0.92
4	0.97		0.95	0.96	0.81	0.83	0.87	0.89	0.99	1.13
5	1.13		1.19	1.17	1.01	0.93	0.90	1.05	0.92	0.93
6	1.15		1.15	1.14	0.98	0.95	0.82	0.83	0.98	1.07
7	1.06		0.98	1.02	1.05	1.04	0.85	0.86	1.02	1.03
8	0.97		0.93	0.93	0.95	0.99	0.76	0.92	0.95	0.96
9	1.07		1.02	1.01	0.86	0.85	0.93	1.09	1.05	1.03

^a^The simulations are conducted under the null hypothesis of no association and no imprinting based on 10,000 replicates for 90 and 150 pedigrees at 1% significance level, respectively

**Fig. 2:**
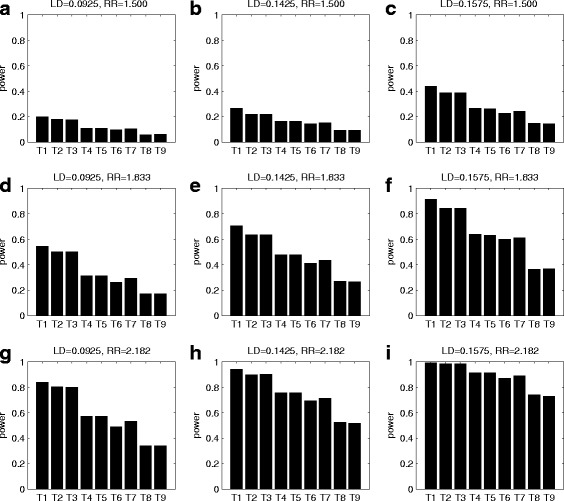
Simulated powers of all the test statistics. The test statistics are T1: GDTI, T2: MCGDTI_T_, T3: MCGDTI_E_, T4: MCGDT-ME_T_, T5: MCGDT-ME_E_, T6: GDT-ME, T7: GDT, T8: MCPDTI_T_ and T9: MCPDTI_E_. The simulations are conducted under complete imprinting effect model at 1% significance level based on 10,000 replicates for 150 pedigrees when LD = 0.092,5, 0.142,5, and 0.157,5, and RR = 1.500, 1.833 and 2.182, respectively. The first 5 statistics are proposed tests, while the remaining 4 are existing tests. **a** LD = 0.092,5 and RR = 1.500; **b** LD = 0.142,5 and RR = 1.500; **c** LD = 0.157,5 and RR = 1.500; **d** LD = 0.092,5 and RR = 1.833; **e** LD = 0.142,5 and RR = 1.833; **f** LD = 0.157,5 and RR = 1.833; **g** LD= 0.092,5 and RR = 2.182; **h** LD = 0.142,5 and RR = 2.182; **i** LD = 0.157,5 and RR = 2.182

**Fig. 3:**
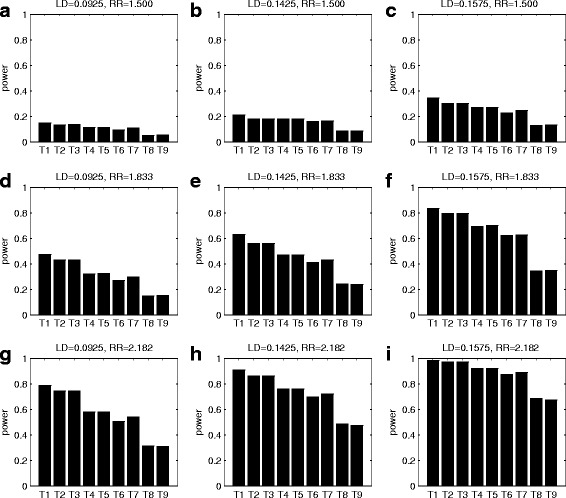
Simulated powers of all the test statistics. The test statistics are T1: GDTI, T2: MCGDTI_T_, T3: MCGDTI_E_, T4: MCGDT-ME_T_, T5: MCGDT-ME_E_, T6: GDT-ME, T7: GDT, T8: MCPDTI_T_ and T9: MCPDTI_E_. The simulations are conducted under incomplete imprinting effect model at 1% significance level based on 10,000 replicates for 150 pedigrees when LD = 0.092,5, 0.142,5, and 0.157,5, and RR = 1.500, 1.833 and 2.182, respectively. The first 5 statistics are proposed tests, while the remaining 4 are existing tests. **a** LD = 0.092,5 and RR = 1.500; **b** LD = 0.142,5 and RR = 1.500; **c** LD = 0.157,5 and RR = 1.500; **d** LD = 0.092,5 and RR = 1.833; **e** LD = 0.142,5 and RR = 1.833; **f** LD = 0.157,5 and RR = 1.833; **g** LD = 0.092,5 and RR = 2.182; **h** LD = 0.142,5 and RR = 2.182; **i** LD = 0.157,5 and RR = 2.182

**Fig. 4:**
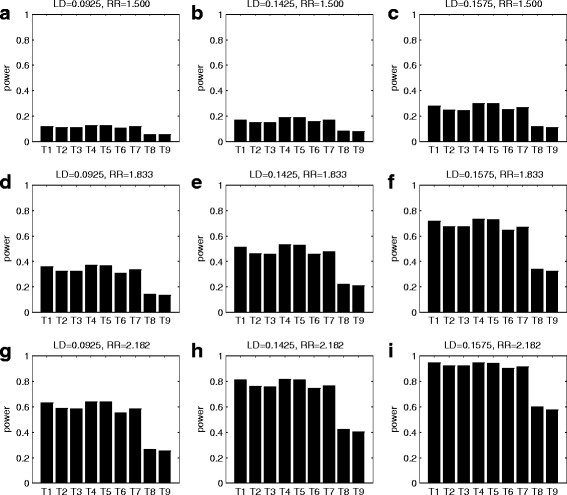
Simulated powers of all the test statistics. The test statistics are T1: GDTI, T2: MCGDTI_T_, T3: MCGDTI_E_, T4: MCGDT-ME_T_, T5: MCGDT-ME_E_, T6: GDT-ME, T7: GDT, T8: MCPDTI_T_ and T9: MCPDTI_E_. The simulations are conducted under no imprinting effect model at 1% significance level based on 10,000 replicates for 150 pedigrees when LD = 0.092,5, 0.142,5, and 0.157,5, and RR = 1.500, 1.833 and 2.182, respectively. The first 5 statistics are proposed tests, while the remaining 4 are existing tests. **a** LD = 0.092,5 and RR = 1.500; **b** LD = 0.142,5 and RR = 1.500; **c** LD = 0.157,5 and RR = 1.500; **d** LD = 0.092,5 and RR = 1.833; **e** LD = 0.142,5 and RR = 1.833; **f** LD = 0.157,5 and RR = 1.833; **g** LD = 0.092,5 and RR = 2.182; **h** LD = 0.142,5 and RR = 2.182; **i** LD = 0.157,5 and RR = 2.182

Figures 2, 3 and 4 give the simulated powers of GDTI, MCGDTI_T_, MCGDTI_E_, MCGDT-ME_T_, MCGDT-ME_E_, GDT-ME, GDT, MCPDTI_T_ and MCPDTI_E_ based on 150 pedigrees at the 1% significance level under complete, incomplete and no imprinting effect models for different LD and RR values, respectively. The first 5 statistics are proposed tests, while the remaining four are existing tests. Additional file 1: Figures S1 - S3 show the corresponding simulated powers of all the methods based on 90 pedigrees. From the figures, we find that the powers of MCGDTI, MCGDT-ME and MCPDTI based on the true marker allele frequencies are very close to those based on the estimated marker allele frequencies (MCGDTI_T_ vs MCGDTI_E_, MCGDT-ME_T_ vs MCGDT-ME_E_, and MCPDTI_T_ vs MCPDTI_E_), respectively. MCGDTI_T_ and MCGDTI_E_ can recapture much of the missing information, which are a little less powerful than GDTI for complete pedigree data. The existing MCPDTI test performs the worst even though it is constructed for testing association when imprinting effects are taken into consideration. On the other hand, MCGDT-ME, GDT-ME and GDT, though without accounting for imprinting, can be used for testing association even when imprinting effects exist. Moreover, they outperform MCPDTI substantially. It is probably due to the fact that MCGDT-ME, GDT-ME and GDT consider genotype differences between all discordant relative pairs, thus utilizing much more information than first-degree relative pairs used by MCPDTI. In Fig. 2 under complete imprinting effect model, when the LD and RR values are fixed, the proposed GDTI (assuming the data are complete) and MCGDTI statistics have higher powers than all the other test statistics. GDT (based on the IBD information) has better performance than GDT-ME, which is the result similar to that in Chen et al. [15]. When the LD value changes from 0.092,5 to 0.157,5 and RR is unchanged, or the LD value is fixed and RR increases from 1.500 to 2.182, all the powers become larger and larger. The results in Fig. 3 under incomplete imprinting effect model are similar to those in Fig. 2. Figure 4 shows the performance of various tests under the no imprinting effect model. The proposed MCGDT-ME outperforms all the existing methods. MCGDTI is a bit less powerful than MCGDT-ME, as expected, and it has a similar performance to GDT-ME and GDT. By comparing the results in Figs. 2, 3 and 4, we find that when the imprinting effect model changes from complete model to incomplete one (i.e. the degree of imprinting effects decreases), the powers of the GDTI and MCGDTI are smaller and smaller. GDTI and MCGDTI attain the least powers under the no imprinting effect model. Finally, the powers of all the methods based on 150 pedigrees are higher than those based on 90 pedigrees (Fig. 2 vs Additional file 1: Figure S1, Fig. 3 vs Additional file 1: Figure S2, and Fig. 4 vs Additional file 1: Figure S3), respectively.

### Application to RA data

We apply our proposed methods to the RA dataset from North American Rheumatoid Arthritis Consortium [20], which is made available from Genetic Analysis Workshop 15 [23]. It has been approved by the providers of the RA data. In this dataset, a total of 757 pedigrees and 8017 individuals were collected, and 5407 autosomal single nucleotide polymorphisms (SNPs) were used. It should be noted that the genotypes of about 80% individuals are missing at these SNPs and thus the proposed MCGDTI (not GDTI) and MCGDT-ME methods are applied. To compare the performance of the proposed tests with the existing methods, we also implement the GDT-ME, GDT and MCPDTI methods in this real data analysis. On the other hand, note that there are 73 pedigree members with unknown affection statuses in this dataset. In addition, we use the existing Monte Carlo pedigree parental-asymmetry test (MCPPAT) to test if imprinting is present [18].

We use the following quality control rules to filter the data. First, a pedigree to be included has at least one affected nonfounder. Second, we delete pedigrees with stepfamilies. Finally, if the proportion of the individuals with missing genotypes among all the members in a pedigree is more than 50% based on the first SNP on Chromosome 1, then we exclude this pedigree. This can avoid the large variability on estimation created by pedigrees with high proportions of missingness. To this end, we get 246 pedigrees with 1109 individuals. Among them, there are 11 individuals with the affection statuses being unavailable and we treat them as unaffected. We use all the available individuals (1992 individuals) in this dataset to estimate the marker allele frequencies, not just using the available founders, due to the large proportion of the individuals with missing genotypes in this dataset. Then, we calculate the values and the corresponding p-values of all the test statistics based on the estimated allele frequencies and 246 selected pedigrees. The significance level is fixed at *α*= 5%, and Bonferroni correction would test each individual hypothesis at the significance level of *α* ^′^= 0.05/5407 = 9.247,3 × 10^−6^, based on 5407 SNPs. The MC size for MCGDTI, MCGDT-ME, MCPDTI and MCPPAT is set to be 50.

The corresponding results of MCGDTI and MCGDT-ME at the significance level of *α*=5%, with Bonferroni correction based on the p-values of these methods are shown in Table 4. From the table, MCGDTI identifies 3 SNPs statistically significantly associated with RA, which cannot be found by MCGDT-ME. Further, the 3 SNPs identified by MCGDTI cannot be detected by GDT-ME, GDT and MCPDTI, and the corresponding contingency tables are the same as Table 4, which are not shown for brevity. The results from this real data application demonstrate a gain in information through incorporating imprinting effects (compared to MCGDT-ME), through making use of partially genotyped pedigrees (compared to GDT-ME and GDT), and through including the genotype differences between beyond first-degree relatives (compared to MCPDTI). In addition, we list the p-values of the association tests MCGDTI, MCGDT-ME, GDT-ME, GDT, MCPDTI and the imprinting test MCPPAT at these 3 SNPs in Additional file 1: Table S1. From the p-values of MCPPAT in this table, there are statistically significant imprinting effects at the 3 SNPs on RA, which may be why MCGDTI is more powerful than the other test statistics.

**Table 4:** Contingency table showing MCGDTI and MCGDT-ME results of application to RA data at *α’* = 9.247,3 × 10^−6a^

	P_MCGDT-ME_ < α’	P_MCGDT-ME_ ≥ α’	Total
P_MCGDTI_ < α’	0	3	3
P_MCGDTI_ ≥ α’	0	5404	5404
Total	0	5407	5407

^a^P_test_ denotes the p-value of the test

## Discussion

In this article, based on a novel decomposition of the genotype score of an individual according to the paternal or maternal source of an allele, we develop the GDTI test to test for association incorporating imprinting for complete pedigrees without missing genotypes. Then, using a MC sampling and estimation scheme, we extend GDTI and GDT-ME, and respectively develop MCGDTI and MCGDT-ME to deal with incomplete pedigrees, in which some individuals’ genotypes are unavailable. Compared to PDTI and MCPDTI, GDTI and MCGDTI make use of the genotype differences between all discordant relative pairs, including beyond first-degree relatives. Simulation results indicate that GDTI, MCGDTI and MCGDT-ME control the size well under the null hypothesis of no association and no imprinting. As for the simulated powers, under complete and incomplete imprinting effect models, our proposed GDTI and MCGDTI methods by considering the information on imprinting effects and all discordant relative pairs outperform all the existing test statistics and MCGDTI can recapture much of the missing information. The application to the RA dataset also demonstrates the advantage of MCGDTI over other methods. Further, in this article, we demonstrate that, the existing GDT-ME and the proposed MCGDT-ME, although not constructed under imprinting, can be used for testing association even when the effects exist. Moreover, we propose the MCGDT-ME test to handle incomplete pedigree data with missing genotypes, and the test is found to perform better than GDT-ME in simulation studies.

One of the major reasons for using within-family tests (e.g. GDT-ME and GDT) for association is their robustness to PS. On the other hand, note that MCGDTI, MCGDT-ME and MCPDTI need the MC sampling and estimation scheme to infer missing genotypes in pedigrees, which requires these pedigrees from a homogenous population. To investigate the performance of the proposed test statistics in the presence of PS, we consider a population consisting of two subpopulations and conduct the following simulation study. The parameters are set to be the same as those in Chen et al. [15]. Specifically, suppose that a disease susceptibility locus and a marker locus are in complete linkage but in linkage equilibrium and both allele frequencies *P*_*D*_ and ${P}_{M_1}$ are taken to be 0.1 (0.5) in the first (second) subpopulation. The penetrances *f*_2_, *f*_10_, *f*_01_ and *f*_0_ of genotypes *D*/*D*, *D*/*d*, *d*/*D* and *d*/*d* are assumed to be 0.45, 0.30, 0.30 and 0.20 in both subpopulations, respectively. In MCGDTI, MCGDT-ME and MCPDTI, the allele frequency ${P}_{M_1}$ is estimated by genotyped founders from all the collected pedigrees, by assuming that they came from a single population, which may cause biases in the estimation of ${P}_{M_1}$. Two simulation scenarios of pedigree structure or level of genotypic missingness are considered. In the first scenario, 150 pedigrees (50 two-generation families, 50 three-generation pedigrees and 50 four-generation pedigrees with the pedigree structures listed in Fig. 1) are sampled from each subpopulation and the only difference between two subpopulations is allele frequencies *P*_*D*_ and ${P}_{M_1}$. In the second scenario, 200 pedigrees (100 two-generation families and 100 three-generation pedigrees with the pedigree structures listed in Fig. 1) are simulated from the first subpopulation and 100 four-generation pedigrees with the pedigree structure listed in Fig. 1 are generated from the second subpopulation, where these two subpopulations are very different from each other in pedigree structure and level of genotypic missingness. Then, the resulting total sample size of pedigrees is 300 for each simulation scenario. Other simulation settings are the same as those in the Simulation settings subsection. The simulated size results of GDTI, MCGDTI, MCGDT-ME, GDT-ME, GDT and MCPDTI are shown in Table 5. From the table, we find that all the proposed test statistics control the size well under the PS models, while the size of the existing MCPDTI test is a little inflated.

**Table 5:** Empirical size (in percentage (%)) of GDTI, MCGDTI, MCGDT-ME, GDT-ME, GDT and MCPDTI in the presence of population stratification^a^

Scenario	Complete data	Incomplete data				
	GDTI	MCGDTI	MCGDT-ME	GDT-ME	GDT	MCPDTI
1	1.14	1.16	1.04	1.14	1.16	1.69
2	1.07	1.01	0.97	1.03	1.09	1.24

^a^The simulations are conducted under the null hypothesis of no association and no imprinting based on 10,000 replicates at 1% significance level

Just like the genotypes of some members in the collected pedigrees may be missing, it is also common in practice that the affection statuses of some individuals in the pedigrees may be unavailable. As mentioned in the real data application subsection, one way to deal with these individuals is to treat them as unaffected. To investigate if this influences the validity of the proposed test statistics, we conduct a few simulation studies. The simulation results show that the proposed methods are still valid to test for association by handling the missing affection status in this way (data not shown). However, this may impact their test powers under alternative hypotheses and we will carry out some simulation studies to check it in our future work.

Like other methods, our proposed GDTI and MCGDTI have their own limitations. In this article, we only consider using an empirical moment estimate based on large sample theory to estimate the variances of the numerators of GDTI and MCGDTI, while we do not propose the corresponding tests based on the variance estimates from the IBD information. This is because even though the IBD information between two alleles for the pair of allele scores (${X}_{ij}^{(p)}$, ${X}_{ik}^{(p)}$), (${X}_{ij}^{(p)}$, ${X}_{ik}^{(m)}$), (${X}_{ij}^{(m)}$, ${X}_{ik}^{(p)}$) or (${X}_{ij}^{(m)}$, ${X}_{ik}^{(m)}$) of the *j* ^th^ and *k* ^th^ individuals in the *i* ^th^ pedigree is obtained, two allele scores in this pair may be different from each other for GDTI and MCGDTI and thus we cannot estimate the corresponding variance based on the IBD information, which is different from GDT (the details refer to Appendix B in Chen et al. [15]). For example, we consider a two-generation family in which the genotypes of the unaffected parents and the affected child are *M*_1_ *M*_2_, *M*_1_ *M*_2_ and *M*_1_ *M*_1_, respectively. Then, when we compare the allele scores of the unaffected father and the affected child, the allele scores of the father and the child are respectively ${X}_F^{(p)}={X}_F^{(m)}=$ 0.5 and ${X}_C^{(p)}={X}_C^{(m)}=$ 1, which are different from each other. Fortunately, from our simulation study, MCGDTI for incomplete pedigrees even has the similar power to GDT under the no imprinting effect model, and is more powerful than GDT under the imprinting effect models.

We should mention that, because of utilizing the genotype differences between all discordant relative pairs, the requirement for a pedigree to be included is that this pedigree should have at least one affected and one unaffected individuals. In addition, GDTI and MCGDTI do not take account of the covariates in analysis, which may cause the dependences between individuals within a family, even though under the null hypothesis of no association. This may be handled from the quasi-likelihood for a conditional logistic regression model [15, 24, 25]. So, our future work is to incorporate the covariates into GDTI and MCGDTI.

## Conclusions

Under complete and incomplete imprinting effect models, our proposed GDTI and MCGDTI methods, by considering the information on imprinting effects and all discordant relative pairs within each pedigree, outperform all the existing test statistics and MCGDTI can recapture much of the missing information. Therefore, MCGDTI is recommended in practice.

## Additional files

Additional file 1: Appendix. Construction of the GDTI test statistic. **Table S1.** P-values of the test statistics applied to RA data at 3 SNPs with *P*_*MCGDTI*_< 9.247,3 × 10^−6^. **Figures S1 - S3.** Simulated powers of all the test statistics. The test statistics are T1: GDTI, T2: MCGDTI_T_, T3: MCGDTI_E_, T4: MCGDT-ME_T_, T5: MCGDT-ME_E_, T6: GDT-ME, T7: GDT, T8: MCPDTI_T_ and T9: MCPDTI_E_. The simulations are conducted under complete, incomplete and no imprinting effect models at 1% significance level based on 10,000 replicates for 90 pedigrees when LD = 0.092,5, 0.142,5, and 0.157,5, and RR = 1.500, 1.833 and 2.182, respectively. The first 5 statistics are proposed tests, while the remaining 4 are existing tests. (PDF 76 kb)

## Abbreviations

GDT: Generalized disequilibrium test with the variance estimated based on the information on kinship coefficients when identity by descent is unknown
GDTI: Generalized disequilibrium test with imprinting
GDT-ME: Generalized disequilibrium test based on the moment estimate of the variance
IBD: Identity by descent
LD: Linkage disequilibrium
MC: Monte Carlo
MCGDTI: Monte Carlo GDTI
MCGDT-ME: Monte Carlo GDT-ME
MCPDTI: Monte Carlo pedigree disequilibrium test with imprinting
MCPPAT: Monte Carlo pedigree parental-asymmetry test
PDTI: Pedigree disequilibrium test with imprinting
PS: Population stratification
RA: Rheumatoid arthritis
RR: Relative risk
SNP: Single nucleotide polymorphism
