# Supplementary material for: Generalized disequilibrium test for association in qualitative traits incorporating imprinting effects based on extended pedigrees
Source: BMC Genet. 2017 Oct 16;18:90. doi: 10.1186/s12863-017-0560-0 (PMC5644153; doi:10.1186/s12863-017-0560-0)
Supplement: Supplementary file 1 — Construction of the GDTI test statistic. Table S1. P-values of the test statistics applied to RA data at 3 SNPs with P MCGDTI< 9.247,3 × 10−6. Figures S1 - S3. Simulated powers of all the test statistics. The test statistics are T1: GDTI, T2: MCGDTIT, T3: MCGDTIE, T4: MCGDT-MET, T5: MCGDT-MEE, T6: GDT-ME, T7: GDT, T8: MCPDTIT and T9: MCPDTIE. The simulations are conducted under complete, incomplete and no imprinting effect models at 1% significance level based on 10,000 replicates for 90 pedigrees when LD = 0.092,5, 0.142,5, and 0.157,5, and RR = 1.500, 1.833 and 2.182, respectively. The first 5 statistics are proposed tests, while the remaining 4 are existing tests. (PDF 76 kb) [file 12863_2017_560_MOESM1_ESM.pdf]

Additional file 1 for "Generalized disequilibrium test for association in qualitative traits incorporating imprinting effects based on extended pedigrees"

Jian-Long Li[1,2], Peng Wang[1], Wing Kam Fung[3*] and Ji-Yuan Zhou[1*]

[1]State Key Laboratory of Organ Failure Research, Ministry of Education, and Guangdong Provincial Key Laboratory of Tropical Disease Research, Department of Biostatistics, School of Public Health, Southern Medical University, Guangzhou, China

[2]State Key Laboratory of Ophthalmology, Zhongshan Ophthalmic Center, Sun Yat-sen University, Guangzhou, China

[3]Department of Statistics and Actuarial Science, The University of Hong Kong, Hong Kong, China

Appendix

Construction of the GDTI test statistic

We only focus on the likelihood of a single pedigree. Suppose that in the ith pedigree, there are N_i individuals in total, among which the first A_i individuals are affected and the other U_i individuals are unaffected. We use the following logistic regression to model the association between the disease status Y_{ij} and the allele scores $X_{ij}^{(p)}$ and $X_{ij}^{(m)}$,

$$\log \frac{P(Y_{ij}=1)}{1-P(Y_{ij}=1)} = \beta_0 + \beta_p X_{ij}^{(p)} + \beta_m X_{ij}^{(m)},$$

where Y_{ij}, $X_{ij}^{(p)}$ and $X_{ij}^{(m)}$ are respectively the disease status and the paternal and maternal allele scores of the jth individual in the ith pedigree. The likelihood that the first A_i individuals are affected, conditional on the fact that there are A_i affected individuals in total in the ith pedigree, is

$$\begin{aligned}
P\left(\sum_{j=1}^{A_i} Y_{ij} = A_i \middle| \sum_{j=1}^{N_i} Y_{ij} = A_i\right) &= \frac{P\left(\sum_{j=1}^{A_i} Y_{ij} = A_i\right)}{\sum_{s_l} P\left(\sum_{j\in s_l} Y_{ij} = A_i\right)} \\
&= \frac{\exp\left(A_i\beta_0 + \sum_{j=1}^{A_i} \beta_p X_{ij}^{(p)} + \sum_{j=1}^{A_i} \beta_m X_{ij}^{(m)}\right)}{\sum_{s_l} \exp\left(A_i\beta_0 + \sum_{j\in s_l} \beta_p X_{ij}^{(p)} + \sum_{j\in s_l} \beta_m X_{ij}^{(m)}\right)} \\
&= \frac{\exp\left(\sum_{j=1}^{A_i} \beta_p X_{ij}^{(p)} + \sum_{j=1}^{A_i} \beta_m X_{ij}^{(m)}\right)}{\sum_{s_l} \exp\left(\sum_{j\in s_l} \beta_p X_{ij}^{(p)} + \sum_{j\in s_l} \beta_m X_{ij}^{(m)}\right)} \\
&= \frac{\exp\left[\sum_{j=1}^{A_i} \left(\beta_p X_{ij}^{(p)} + \beta_m X_{ij}^{(m)}\right)\right]}{\sum_{s_l} \exp\left[\sum_{j\in s_l} \left(\beta_p X_{ij}^{(p)} + \beta_m X_{ij}^{(m)}\right)\right]}
\end{aligned}$$

$$\times \frac{\exp\left[-\frac{A_i}{U_i}\left(\beta_p X_{i(A_i+1)}^{(p)} + \beta_m X_{i(A_i+1)}^{(m)} + \ldots + \beta_p X_{iN_i}^{(p)} + \beta_m X_{iN_i}^{(m)}\right)\right]}{\exp\left[-\frac{A_i}{U_i}\left(\beta_p X_{i(A_i+1)}^{(p)} + \beta_m X_{i(A_i+1)}^{(m)} + \ldots + \beta_p X_{iN_i}^{(p)} + \beta_m X_{iN_i}^{(m)}\right)\right]}$$

$$= \frac{\exp\left\{\frac{1}{U_i}\sum_{j=1}^{A_i}\sum_{k=A_i+1}^{N_i}\left[\left(X_{ij}^{(p)} - X_{ik}^{(p)}\right)\beta_p + \left(X_{ij}^{(m)} - X_{ik}^{(m)}\right)\beta_m\right]\right\}}{\sum_{s_l}\exp\left\{\frac{1}{U_i}\sum_{j\in s_l}\sum_{k=A_i+1}^{N_i}\left[\left(X_{ij}^{(p)} - X_{ik}^{(p)}\right)\beta_p + \left(X_{ij}^{(m)} - X_{ik}^{(m)}\right)\beta_m\right]\right\}}.$$

The log-likelihood function is

$$l_i = \frac{1}{U_i}\sum_{j=1}^{A_i}\sum_{k=A_i+1}^{N_i}\left[\left(X_{ij}^{(p)} - X_{ik}^{(p)}\right)\beta_p + \left(X_{ij}^{(m)} - X_{ik}^{(m)}\right)\beta_m\right]$$
$$- \log\sum_{s_l}\exp\left\{\frac{1}{U_i}\sum_{j\in s_l}\sum_{k=A_i+1}^{N_i}\left[\left(X_{ij}^{(p)} - X_{ik}^{(p)}\right)\beta_p + \left(X_{ij}^{(m)} - X_{ik}^{(m)}\right)\beta_m\right]\right\}.$$

Then,

$$\frac{\partial l_i}{\partial \beta_p} = \frac{1}{U_i}\sum_{j=1}^{A_i}\sum_{k=A_i+1}^{N_i}\left(X_{ij}^{(p)} - X_{ik}^{(p)}\right)$$

$$- \frac{\sum_{s_l}\left\{\exp\left[\frac{1}{U_i}\sum_{j\in s_l}\sum_{k=A_i+1}^{N_i}\left(\left(X_{ij}^{(p)} - X_{ik}^{(p)}\right)\beta_p + \left(X_{ij}^{(m)} - X_{ik}^{(m)}\right)\beta_m\right)\right] \cdot \left(\frac{1}{U_i}\sum_{j\in s_l}\sum_{k=A_i+1}^{N_i}\left(X_{ij}^{(p)} - X_{ik}^{(p)}\right)\right)\right\}}{\sum_{s_l}\exp\left\{\frac{1}{U_i}\sum_{j\in s_l}\sum_{k=A_i+1}^{N_i}\left[\left(X_{ij}^{(p)} - X_{ik}^{(p)}\right)\beta_p + \left(X_{ij}^{(m)} - X_{ik}^{(m)}\right)\beta_m\right]\right\}},$$

$$\frac{\partial l_i}{\partial \beta_m} = \frac{1}{U_i}\sum_{j=1}^{A_i}\sum_{k=A_i+1}^{N_i}\left(X_{ij}^{(m)} - X_{ik}^{(m)}\right)$$

$$- \frac{\sum_{s_l}\left\{\exp\left[\frac{1}{U_i}\sum_{j\in s_l}\sum_{k=A_i+1}^{N_i}\left(\left(X_{ij}^{(p)} - X_{ik}^{(p)}\right)\beta_p + \left(X_{ij}^{(m)} - X_{ik}^{(m)}\right)\beta_m\right)\right] \cdot \left(\frac{1}{U_i}\sum_{j\in s_l}\sum_{k=A_i+1}^{N_i}\left(X_{ij}^{(m)} - X_{ik}^{(m)}\right)\right)\right\}}{\sum_{s_l}\exp\left\{\frac{1}{U_i}\sum_{j\in s_l}\sum_{k=A_i+1}^{N_i}\left[\left(X_{ij}^{(p)} - X_{ik}^{(p)}\right)\beta_p + \left(X_{ij}^{(m)} - X_{ik}^{(m)}\right)\beta_m\right]\right\}},$$

$$\frac{\partial^2 l_i}{\partial \beta_p^2} = -\frac{\begin{array}{c}\sum_{s_l}\left\{\exp\left[\frac{1}{U_i}\sum_{j\in s_l}\sum_{k=A_i+1}^{N_i}\left(\left(X_{ij}^{(p)}-X_{ik}^{(p)}\right)\beta_p+\left(X_{ij}^{(m)}-X_{ik}^{(m)}\right)\beta_m\right)\right]\right.\\ \left.\cdot\left(\frac{1}{U_i}\sum_{j\in s_l}\sum_{k=A_i+1}^{N_i}\left(X_{ij}^{(p)}-X_{ik}^{(p)}\right)\right)^2\right\}\end{array}}{\sum_{s_l}\exp\left\{\frac{1}{U_i}\sum_{j\in s_l}\sum_{k=A_i+1}^{N_i}\left[\left(X_{ij}^{(p)}-X_{ik}^{(p)}\right)\beta_p+\left(X_{ij}^{(m)}-X_{ik}^{(m)}\right)\beta_m\right]\right\}}$$

$$+\frac{\begin{array}{c}\left\{\sum_{s_l}\left[\exp\left(\frac{1}{U_i}\sum_{j\in s_l}\sum_{k=A_i+1}^{N_i}\left(\left(X_{ij}^{(p)}-X_{ik}^{(p)}\right)\beta_p+\left(X_{ij}^{(m)}-X_{ik}^{(m)}\right)\beta_m\right)\right)\right.\right.\\ \left.\left.\cdot\left(\frac{1}{U_i}\sum_{j\in s_l}\sum_{k=A_i+1}^{N_i}\left(X_{ij}^{(p)}-X_{ik}^{(p)}\right)\right)\right]\right\}^2\end{array}}{\left\{\sum_{s_l}\exp\left[\frac{1}{U_i}\sum_{j\in s_l}\sum_{k=A_i+1}^{N_i}\left(\left(X_{ij}^{(p)}-X_{ik}^{(p)}\right)\beta_p+\left(X_{ij}^{(m)}-X_{ik}^{(m)}\right)\beta_m\right)\right]\right\}^2},$$

$$\frac{\partial^2 l_i}{\partial \beta_m^2} = -\frac{\begin{array}{c}\sum_{s_l}\left\{\exp\left[\frac{1}{U_i}\sum_{j\in s_l}\sum_{k=A_i+1}^{N_i}\left(\left(X_{ij}^{(p)}-X_{ik}^{(p)}\right)\beta_p+\left(X_{ij}^{(m)}-X_{ik}^{(m)}\right)\beta_m\right)\right]\right.\\ \left.\cdot\left(\frac{1}{U_i}\sum_{j\in s_l}\sum_{k=A_i+1}^{N_i}\left(X_{ij}^{(m)}-X_{ik}^{(m)}\right)\right)^2\right\}\end{array}}{\sum_{s_l}\exp\left\{\frac{1}{U_i}\sum_{j\in s_l}\sum_{k=A_i+1}^{N_i}\left[\left(X_{ij}^{(p)}-X_{ik}^{(p)}\right)\beta_p+\left(X_{ij}^{(m)}-X_{ik}^{(m)}\right)\beta_m\right]\right\}}$$

$$+\frac{\begin{array}{c}\left\{\sum_{s_l}\left[\exp\left(\frac{1}{U_i}\sum_{j\in s_l}\sum_{k=A_i+1}^{N_i}\left(\left(X_{ij}^{(p)}-X_{ik}^{(p)}\right)\beta_p+\left(X_{ij}^{(m)}-X_{ik}^{(m)}\right)\beta_m\right)\right)\right.\right.\\ \left.\left.\cdot\left(\frac{1}{U_i}\sum_{j\in s_l}\sum_{k=A_i+1}^{N_i}\left(X_{ij}^{(m)}-X_{ik}^{(m)}\right)\right)\right]\right\}^2\end{array}}{\left\{\sum_{s_l}\exp\left[\frac{1}{U_i}\sum_{j\in s_l}\sum_{k=A_i+1}^{N_i}\left(\left(X_{ij}^{(p)}-X_{ik}^{(p)}\right)\beta_p+\left(X_{ij}^{(m)}-X_{ik}^{(m)}\right)\beta_m\right)\right]\right\}^2},$$

$$\frac{\partial^2 l_i}{\partial \beta_p \partial \beta_m} = -\frac{\sum_{s_l} \left\{ \exp\left[\frac{1}{U_i} \sum_{j \in s_l} \sum_{k=A_i+1}^{N_i} \left(\left(X_{ij}^{(p)} - X_{ik}^{(p)} \right) \beta_p + \left(X_{ij}^{(m)} - X_{ik}^{(m)} \right) \beta_m \right) \right] \cdot \left(\frac{1}{U_i} \sum_{j \in s_l} \sum_{k=A_i+1}^{N_i} \left(X_{ij}^{(p)} - X_{ik}^{(p)} \right) \right) \left(\frac{1}{U_i} \sum_{j \in s_l} \sum_{k=A_i+1}^{N_i} \left(X_{ij}^{(m)} - X_{ik}^{(m)} \right) \right) \right\}}{\sum_{s_l} \exp\left\{ \frac{1}{U_i} \sum_{j \in s_l} \sum_{k=A_i+1}^{N_i} \left[\left(X_{ij}^{(p)} - X_{ik}^{(p)} \right) \beta_p + \left(X_{ij}^{(m)} - X_{ik}^{(m)} \right) \beta_m \right] \right\}}$$

$$+ \frac{\begin{aligned} &\sum_{s_l} \left\{ \exp\left[\frac{1}{U_i} \sum_{j \in s_l} \sum_{k=A_i+1}^{N_i} \left(\left(X_{ij}^{(p)} - X_{ik}^{(p)} \right) \beta_p + \left(X_{ij}^{(m)} - X_{ik}^{(m)} \right) \beta_m \right) \right] \cdot \left(\frac{1}{U_i} \sum_{j \in s_l} \sum_{k=A_i+1}^{N_i} \left(X_{ij}^{(p)} - X_{ik}^{(p)} \right) \right) \right\} \\ &\cdot \sum_{s_l} \left\{ \exp\left[\frac{1}{U_i} \sum_{j \in s_l} \sum_{k=A_i+1}^{N_i} \left(\left(X_{ij}^{(p)} - X_{ik}^{(p)} \right) \beta_p + \left(X_{ij}^{(m)} - X_{ik}^{(m)} \right) \beta_m \right) \right] \cdot \left(\frac{1}{U_i} \sum_{j \in s_l} \sum_{k=A_i+1}^{N_i} \left(X_{ij}^{(m)} - X_{ik}^{(m)} \right) \right) \right\} \end{aligned}}{\left\{ \sum_{s_l} \exp\left[\frac{1}{U_i} \sum_{j \in s_l} \sum_{k=A_i+1}^{N_i} \left(\left(X_{ij}^{(p)} - X_{ik}^{(p)} \right) \beta_p + \left(X_{ij}^{(m)} - X_{ik}^{(m)} \right) \beta_m \right) \right] \right\}^2},$$

$$\frac{\partial^2 l_i}{\partial \beta_m \partial \beta_p} = -\frac{\sum_{s_l} \left\{ \exp\left[\frac{1}{U_i} \sum_{j \in s_l} \sum_{k=A_i+1}^{N_i} \left(\left(X_{ij}^{(p)} - X_{ik}^{(p)} \right) \beta_p + \left(X_{ij}^{(m)} - X_{ik}^{(m)} \right) \beta_m \right) \right] \cdot \left(\frac{1}{U_i} \sum_{j \in s_l} \sum_{k=A_i+1}^{N_i} \left(X_{ij}^{(m)} - X_{ik}^{(m)} \right) \right) \left(\frac{1}{U_i} \sum_{j \in s_l} \sum_{k=A_i+1}^{N_i} \left(X_{ij}^{(p)} - X_{ik}^{(p)} \right) \right) \right\}}{\sum_{s_l} \exp\left\{ \frac{1}{U_i} \sum_{j \in s_l} \sum_{k=A_i+1}^{N_i} \left[\left(X_{ij}^{(p)} - X_{ik}^{(p)} \right) \beta_p + \left(X_{ij}^{(m)} - X_{ik}^{(m)} \right) \beta_m \right] \right\}}$$

$$
+\frac{\begin{array}{c}\sum_{s_l}\left\{\exp\left[\frac{1}{U_i}\sum_{j\in s_l}\sum_{k=A_i+1}^{N_i}\left(\left(X_{ij}^{(p)}-X_{ik}^{(p)}\right)\beta_p+\left(X_{ij}^{(m)}-X_{ik}^{(m)}\right)\beta_m\right)\right]\right.\\ \left.\cdot\left(\frac{1}{U_i}\sum_{j\in s_l}\sum_{k=A_i+1}^{N_i}\left(X_{ij}^{(m)}-X_{ik}^{(m)}\right)\right)\right\}\\ \cdot\sum_{s_l}\left\{\exp\left[\frac{1}{U_i}\sum_{j\in s_l}\sum_{k=A_i+1}^{N_i}\left(\left(X_{ij}^{(p)}-X_{ik}^{(p)}\right)\beta_p+\left(X_{ij}^{(m)}-X_{ik}^{(m)}\right)\beta_m\right)\right]\right.\\ \left.\cdot\left(\frac{1}{U_i}\sum_{j\in s_l}\sum_{k=A_i+1}^{N_i}\left(X_{ij}^{(p)}-X_{ik}^{(p)}\right)\right)\right\}\end{array}}{\left\{\sum_{s_l}\exp\left[\frac{1}{U_i}\sum_{j\in s_l}\sum_{k=A_i+1}^{N_i}\left(\left(X_{ij}^{(p)}-X_{ik}^{(p)}\right)\beta_p+\left(X_{ij}^{(m)}-X_{ik}^{(m)}\right)\beta_m\right)\right]\right\}^2}.
$$

Under the null hypothesis of no association and no imprinting, we have

$$
\begin{aligned}
D_{i1}=\frac{\partial l_i}{\partial\beta_p}|_{\beta_p=0,\beta_m=0} &= \frac{1}{U_i}\sum_{j=1}^{A_i}\sum_{k=A_i+1}^{N_i}\left(X_{ij}^{(p)}-X_{ik}^{(p)}\right)-\frac{\sum_{s_l}\left[\frac{1}{U_i}\sum_{j\in s_l}\sum_{k=A_i+1}^{N_i}\left(X_{ij}^{(p)}-X_{ik}^{(p)}\right)\right]}{\binom{N_i}{A_i}}\\
&= \frac{1}{U_i}\sum_{j=1}^{A_i}\sum_{k=A_i+1}^{N_i}\left(X_{ij}^{(p)}-X_{ik}^{(p)}\right)-\frac{\frac{1}{U_i}\sum_{s_l}\sum_{j\in s_l}\left(U_iX_{ij}^{(p)}-X_{i(A_i+1)}^{(p)}-\ldots-X_{iN_i}^{(p)}\right)}{\binom{N_i}{A_i}}\\
&= \frac{1}{U_i}\sum_{j=1}^{A_i}\sum_{k=A_i+1}^{N_i}\left(X_{ij}^{(p)}-X_{ik}^{(p)}\right)\\
&\quad-\frac{\frac{1}{U_i}\sum_{s_l}\left[U_i\sum_{j\in s_l}X_{ij}^{(p)}-A_i\left(X_{i(A_i+1)}^{(p)}+\ldots+X_{iN_i}^{(p)}\right)\right]}{\binom{N_i}{A_i}}\\
&= \frac{1}{U_i}\sum_{j=1}^{A_i}\sum_{k=A_i+1}^{N_i}\left(X_{ij}^{(p)}-X_{ik}^{(p)}\right)\\
&\quad-\frac{1}{U_i}\frac{\binom{N_i-1}{A_i-1}U_i\sum_{j=1}^{N_i}X_{ij}-\binom{N_i}{A_i}A_i\left(X_{i(A_i+1)}^{(p)}+\ldots+X_{iN_i}^{(p)}\right)}{\binom{N_i}{A_i}}\\
&= \frac{1}{U_i}\sum_{j=1}^{A_i}\sum_{k=A_i+1}^{N_i}\left(X_{ij}^{(p)}-X_{ik}^{(p)}\right)\\
&\quad-\frac{1}{U_i}\frac{\binom{N_i}{A_i}\frac{A_iU_i}{N_i}\sum_{j=1}^{N_i}X_{ij}^{(p)}-\binom{N_i}{A_i}A_i\left(X_{i(A_i+1)}^{(p)}+\ldots+X_{iN_i}^{(p)}\right)}{\binom{N_i}{A_i}}\\
&= \frac{1}{N_i}\sum_{j=1}^{A_i}\sum_{k=A_i+1}^{N_i}\left(X_{ij}^{(p)}-X_{ik}^{(p)}\right).
\end{aligned}
$$

Similarly, we can get the following equations

$$D_{i2} = \frac{\partial l_i}{\partial \beta_m}|_{\beta_p=0,\beta_m=0} = \frac{1}{N_i}\sum_{j=1}^{A_i}\sum_{k=A_i+1}^{N_i}\left(X_{ij}^{(m)} - X_{ik}^{(m)}\right),$$

$$I_{i11} = -\frac{\partial^2 l_i}{\partial \beta_p^2}|_{\beta_p=0,\beta_m=0} = \frac{\left(\frac{1}{U_i}\right)^2 \sum_{s_l}\left[\sum_{j\in s_l}\sum_{k=A_i+1}^{N_i}\left(X_{ij}^{(p)} - X_{ik}^{(p)}\right)\right]^2}{\binom{N_i}{A_i}} - \left(\frac{A_i}{U_iN_i}\right)^2\left[\sum_{j=1}^{A_i}\sum_{k=A_i+1}^{N_i}\left(X_{ij}^{(p)} - X_{ik}^{(p)}\right)\right]^2,$$

$$I_{i22} = -\frac{\partial^2 l_i}{\partial \beta_m^2}|_{\beta_p=0,\beta_m=0} = \frac{\left(\frac{1}{U_i}\right)^2 \sum_{s_l}\left[\sum_{j\in s_l}\sum_{k=A_i+1}^{N_i}\left(X_{ij}^{(m)} - X_{ik}^{(m)}\right)\right]^2}{\binom{N_i}{A_i}} - \left(\frac{A_i}{U_iN_i}\right)^2\left[\sum_{j=1}^{A_i}\sum_{k=A_i+1}^{N_i}\left(X_{ij}^{(m)} - X_{ik}^{(m)}\right)\right]^2,$$

$$I_{i12} = I_{i21} = -\frac{\partial^2 l_i}{\partial \beta_p \partial \beta_m}|_{\beta_p=0,\beta_m=0} = -\frac{\partial^2 l_i}{\partial \beta_m \partial \beta_p}|_{\beta_p=0,\beta_m=0}$$

$$= \frac{\left(\frac{1}{U_i}\right)^2 \sum_{s_l}\left\{\left[\sum_{j\in s_l}\sum_{k=A_i+1}^{N_i}\left(X_{ij}^{(p)} - X_{ik}^{(p)}\right)\right]\cdot\left[\sum_{j\in s_l}\sum_{k=A_i+1}^{N_i}\left(X_{ij}^{(m)} - X_{ik}^{(m)}\right)\right]\right\}}{\binom{N_i}{A_i}} - \left(\frac{A_i}{U_iN_i}\right)^2\left[\sum_{j=1}^{A_i}\sum_{k=A_i+1}^{N_i}\left(X_{ij}^{(p)} - X_{ik}^{(p)}\right)\right]\left[\sum_{j=1}^{A_i}\sum_{k=A_i+1}^{N_i}\left(X_{ij}^{(m)} - X_{ik}^{(m)}\right)\right].$$

Table S1 P-values of the test statistics applied to RA data at 3 SNPs with $P_{\mathrm{MCGDTI}} < 9.247, 3 \times 10^{-6}$

SNP name	Chr.[a]	$\mathrm{P_{MCGDTI}}$[b]	$\mathrm{P_{MCGDT-ME}}$	$\mathrm{P_{GDT-ME}}$	$\mathrm{P_{GDT}}$	$\mathrm{P_{MCPDTI}}$	$\mathrm{P_{MCPPAT}}$
rs813079	6	2.19×10^{-6}	0.2261	0.0327	0.0303	0.0109	0.0002
rs1488318	6	5.35×10^{-6}	0.0807	0.3813	0.4108	0.1657	0.0027
rs1211375	16	2.17×10^{-6}	0.8597	0.1353	0.1358	0.1155	3.62×10^{-5}

[a]Chr.: Chromosome. [b]P_{test} denotes the p-value of the test.

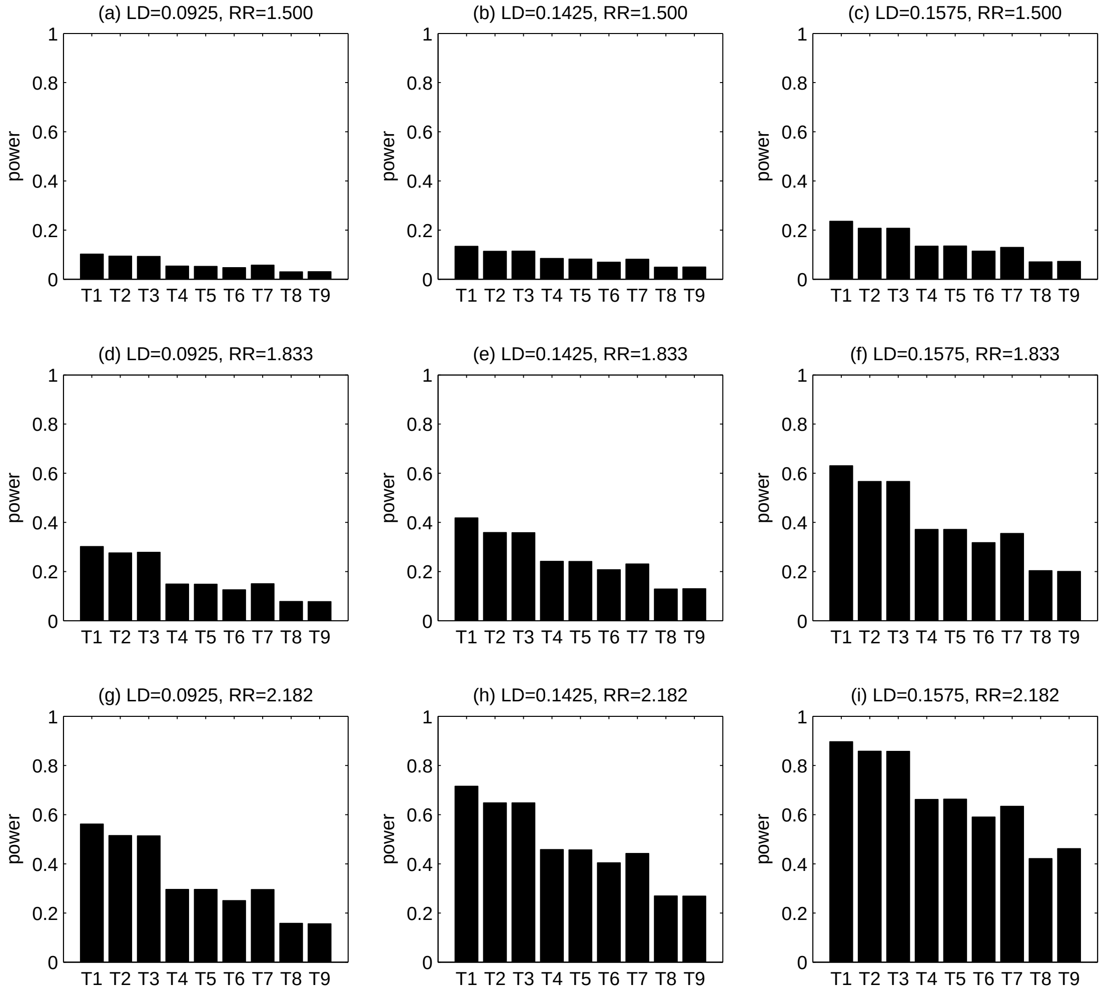

Fig. S1 Simulated powers of all the test statistics. The test statistics are T1: GDTI, T2: $\mathrm{MCGDTI_T}$, T3: $\mathrm{MCGDTI_E}$, T4: $\mathrm{MCGDT\text{-}ME_T}$, T5: $\mathrm{MCGDT\text{-}ME_E}$, T6: GDT-ME, T7: GDT, T8: $\mathrm{MCPDTI_T}$ and T9: $\mathrm{MCPDTI_E}$. The simulations are conducted under complete imprinting effect model at 1% significance level based on 10,000 replicates for 90 pedigrees when LD = 0.092,5, 0.142,5, and 0.157,5, and RR = 1.500, 1.833 and 2.182, respectively. The first 5 statistics are proposed tests, while the remaining 4 are existing tests

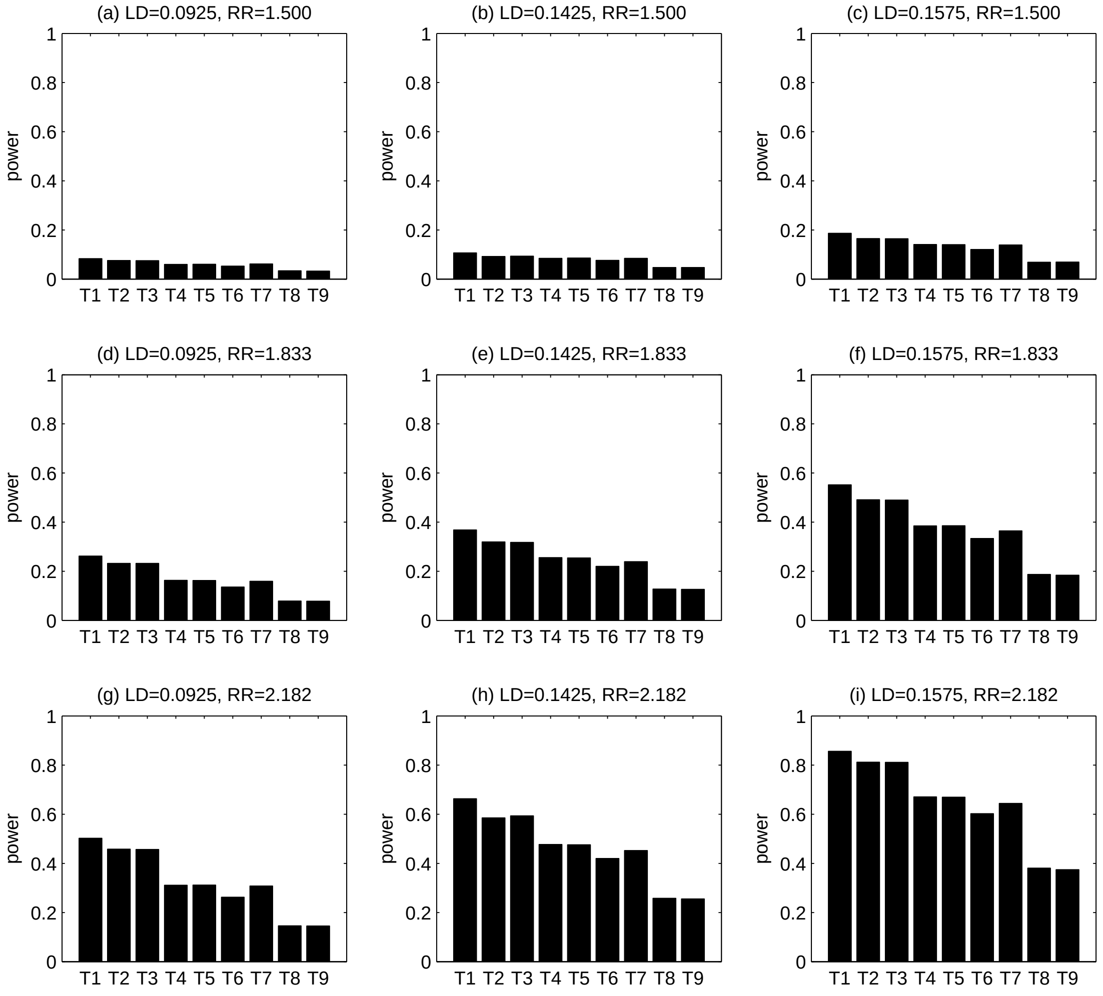

Fig. S2 Simulated powers of all the test statistics. The test statistics are T1: GDTI, T2: $\mathrm{MCGDTI_T}$, T3: $\mathrm{MCGDTI_E}$, T4: $\mathrm{MCGDT\text{-}ME_T}$, T5: $\mathrm{MCGDT\text{-}ME_E}$, T6: GDT-ME, T7: GDT, T8: $\mathrm{MCPDTI_T}$ and T9: $\mathrm{MCPDTI_E}$. The simulations are conducted under incomplete imprinting effect model at 1% significance level based on 10,000 replicates for 90 pedigrees when LD $=$ 0.092,5, 0.142,5, and 0.157,5, and RR $=$ 1.500, 1.833 and 2.182, respectively. The first 5 statistics are proposed tests, while the remaining 4 are existing tests

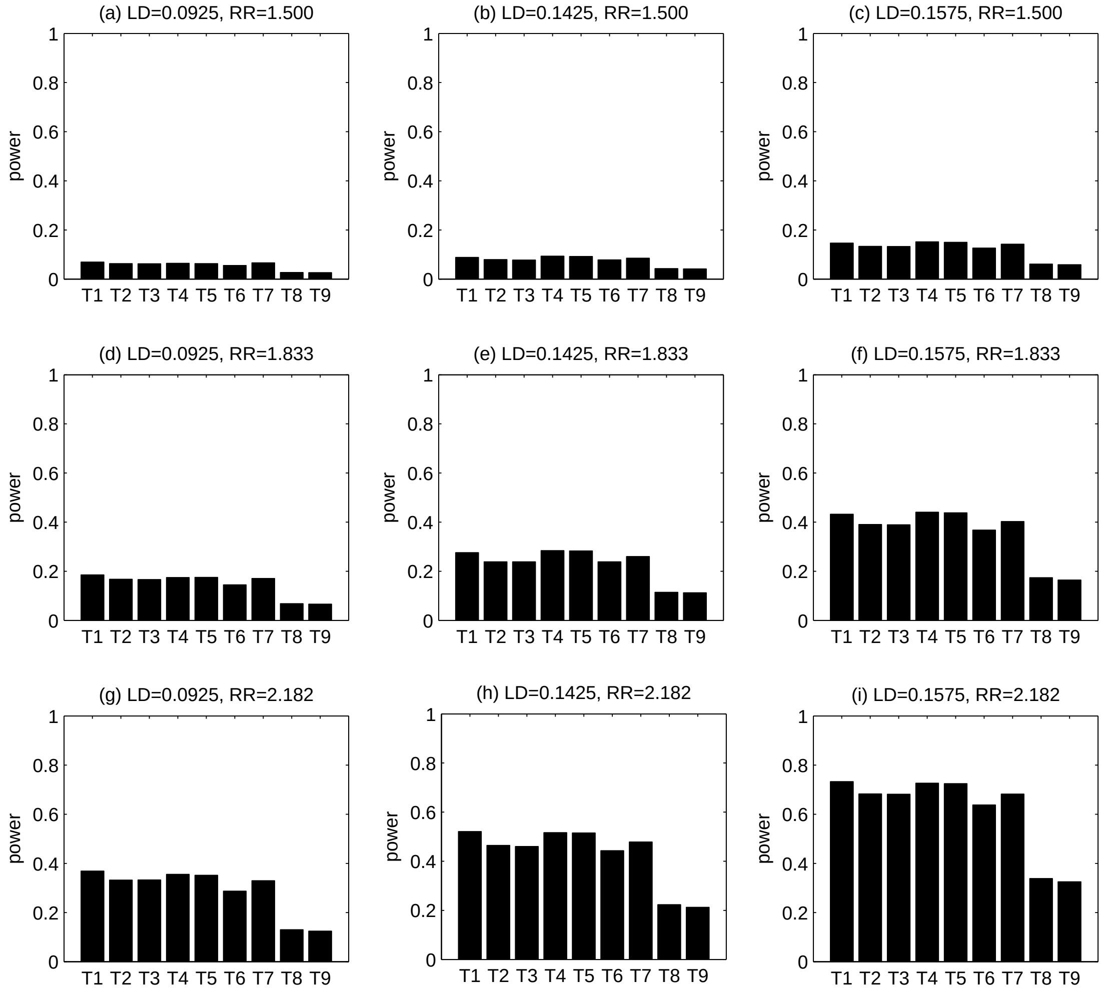

Fig. S3 Simulated powers of all the test statistics. The test statistics are T1: GDTI, T2: $\mathrm{MCGDTI_T}$, T3: $\mathrm{MCGDTI_E}$, T4: $\mathrm{MCGDT\text{-}ME_T}$, T5: $\mathrm{MCGDT\text{-}ME_E}$, T6: GDT-ME, T7: GDT, T8: $\mathrm{MCPDTI_T}$ and T9: $\mathrm{MCPDTI_E}$. The simulations are conducted under no imprinting effect model at 1% significance level based on 10,000 replicates for 90 pedigrees when LD = 0.092,5, 0.142,5, and 0.157,5, and RR = 1.500, 1.833 and 2.182, respectively. The first 5 statistics are proposed tests, while the remaining 4 are existing tests
